# Assessing the psychosocial impact of a vitiligo support group in Ethiopia: A pilot prospective study

**DOI:** 10.1016/j.jdin.2026.05.008

**Published:** 2026-05-18

**Authors:** Feben Messele, Girum Tedla, Sara Ayele, Adane Chernet, Jennifer Roux, Olamide Sonuga Finney, Micayla Wilson, Kristen M. Kelly, Marissa Ericson, Jessica Shiu

**Affiliations:** a Department of Family Medicine, Adventist Health Sonora, Hanford, California; b Hawassa University, Hawassa, Ethiopia; c Department of Dermatology, University of California, Irvine, Irvine, California; d Biostatistics, Epidemiology and Research Design Unit, University of California Irvine, Irvine, California

**Keywords:** depression, PHQ-9, quality of life, vitiligo, vitiligo support groups, VitiQoL

*To the Editor:* Vitiligo is an autoimmune condition that negatively impacts quality of life and often leads to stigmatization, social isolation, and depression.^1^^,^^2^ In East Africa, estimated prevalence of vitiligo is 0.31%, based on Bayesian hierarchical linear mixed models used to compensate for the limited availability of epidemiological data.^3^ A study in Northern Ethiopia reported widespread misconceptions and negative perceptions of vitiligo.^4^ There’s limited research in Ethiopia assessing impact using validated tools.

This is a pilot, prospective, prepost study—approved by the Hawassa University Institutional Review Board in Ethiopia, in collaboration with UC Irvine Department of Dermatology. We assessed baseline and postsupport group changes in quality of life (VitiQoL) and depressive symptoms (PHQ-9) in the Mulu Berhan vitiligo support group participants. Monthly sessions consisted of dermatologist led sessions of vitiligo basics and therapy, a psychiatrist led mental health session, as well as story sharing and self-expression sessions. Repeated-measures analyses were conducted using linear regression with cluster-robust standard errors to account for within-participant correlation arising from repeated observations. This approach yields population-average estimates and is robust to unequal numbers of observations per participant.

Twenty-one patients (53% female) attended at least 1 session, with 4 patients attending 3-4 sessions and 3 patients attending 5-8 sessions (Table I). The average one-way transportation time to each session was 2.7 hours (range 0.25-24). Generalized vitiligo was the most common form (38%), and 76.2% of patients used topical treatments. Notably, 61.9% of participants subjectively reported improvement.

**Table I:** Demographics, VitiQoL, and PHQ-9 among Mulu Berhan vitiligo support group participants in Hawassa, Ethiopia

Study participants	
Participants (*n*)	21 (53% female).
Frequency of participation	• 5-8 sessions attended: 3 • 3-4 sessions attended: 4 • 1-2 sessions attended: 14
Mean age (y)	30.9 (12-57).
Mean 1 way travel time (h)	2.7 (0.25-24)
Education level	
Less than grade 6	8 (38.1%)
High school	8 (38.1%)
College	4 (19%)
Masters	1 (4.8%)
Occupation	
Student	4 (19%)
Unemployed	3 (14.3%)
Employed	13 (61.9%)
Retired	1 (4.8%)
Mode of transportation to support group	
Taxi	8 (38.1%)
Bus	12 (57.1%)
Other	1 (4.8%)
Reported vitiligo subtype	
Focal	6 (28.6%)
Acrofacial	6 (28.6%)
Generalized	8 (38%)
Unspecified	1 (4.8%)
Patient reported outcomes at baseline	
Slowly progressive	5 (23.8%)
Improving	13 (61.9%)
Rapidly progressive	2 (9.5%)
No change	1 (4.8%)
Treatments	
Topical∗	14 (66.7%)
Topical∗ + PO†	2 (9.5%)
PO†	3 (14.3%)
Unspecified	2 (9.5%)
Baseline mean VitiQoL	43.2 (*n* = 19, SD = 21.0)
Baseline mean PHQ-9	5.3 (*n* = 21, SD = 5.0)
Regular support group attendees	Measured at third support group attendance
Presupport group mean VitiQoL	47 (*n* = 7, SD = 26.5)
Postsupport group mean VitiQoL	22.8 (*n* = 7, SD = 5.9)‡
Presupport group mean PHQ-9	4.8 (*n* = 7, SD = 4.9)
Postsupport group mean PHQ-9	1.8 (*n* = 7, SD = 2.6)

∗ Topical denotes use of either clobetasol or mometasone.

† PO denotes use of oral supplements/steroids (such as multivitamins, prednisolone).

‡ Statistically significant change (*P* < .05) using repeated-measures analysis.

At baseline, mean PHQ-9 score 5.3 (*n* = 21, SD = 5.0) and mean VitiQoL score was 43.2 (*n* = 19, SD = 21.0). Repeated-measures analyses demonstrated a statistically significant change in VitiQoL over time. Across repeated attendances (≥3 sessions), VitiQoL total scores decreased significantly, indicating improvement (β = −4.61, *P* = .009; 95% CI: −7.85 to −1.38). This effect remained robust despite unequal numbers of observations per participant and presence of missing data, reflecting a consistent population-level improvement across visits. Variance component estimates indicated substantial between-participant variability in baseline VitiQoL scores, supporting the appropriateness of modeling within-subject correlation. In contrast, repeated-measures analyses of depressive symptoms, assessed using the Patient Health Questionnaire-9 (PHQ-9), did not demonstrate a statistically significant change over time, although scores showed a modest downward trend across attendances (β = −0.37 points per attendance, *P* = .24; 95% CI: −0.99 to 0.24).

To our knowledge, this is the first study to establish and assess impact of a vitiligo support group in Ethiopia using validated tools. The significant decrease in VitiQoL scores indicates a potential benefit of support groups despite long travel times >2h each way. Medication use is a confounding factor for VitiQoL improvement; results may not be solely attributed to the support group. Others have found vitiligo support groups improve Dermatology Life Quality Index scores.^5^ Our findings similarly indicate a beneficial role irrespective of disease burden and logistical challenges in a limited resource setting. Further research involving larger cohorts may give additional insight. Future studies could explore solutions to overcome barriers and incorporate support groups for vitiligo patients in Ethiopia and other resource-limited settings.

### Declaration of generative AI and AI-assisted technologies in the writing process

AI was not used in the composition of this manuscript.

## Conflicts of interest

None disclosed.
